# Inorganic Element Identification and In Vitro Preliminary Evaluation of Three Types of Standardized Black Chokeberry Extracts Against Human Pulmonary Artery Endothelial Cells (HPAECs)

**DOI:** 10.3390/plants14081202

**Published:** 2025-04-12

**Authors:** Valentina Oana Buda, Camelia Oprean, Oana Isabella Gavriliuc, Zorita Diaconeasa, Adina Căta, Daniela Haidu, Daliana Minda, Andreea Păunescu, Cristina Adriana Dehelean, Corina Danciu

**Affiliations:** 1 Faculty of Pharmacy, “Victor Babes” University of Medicine and Pharmacy, E. Murgu Sq., No. 2, 300041 Timisoara, Romania; buda.valentina@umft.ro (V.O.B.); daliana.minda@umft.ro (D.M.); cadehelean@umft.ro (C.A.D.); corina.danciu@umft.ro (C.D.); 2 Research Centre for Pharmaco-Toxicological Evaluation, “Victor Babes” University of Medicine and Pharmacy, E. Murgu Sq., No. 2, 300041 Timisoara, Romania; 3 Research and Processing Center for Medicinal and Aromatic Plants, “Victor Babes” University of Medicine and Pharmacy, E. Murgu Sq., No. 2, 300041 Timisoara, Romania; 4 Center for Drug Data Analysis, Cheminformatics, and the Internet of Medical Things, “Victor Babes” University of Medicine and Pharmacy, E. Murgu Sq., No. 2, 300041 Timisoara, Romania; 5 OncoGen Center for Gene and Cellular Therapies in the Treatment of Cancer, “Pius Brînzeu” County Clinical Emergency Hospital Timișoara, Blvd. Liviu Rebreanu 156, 300723 Timisoara, Romania; gavriliuc.oana@umft.ro; 6 Faculty of Medicine, “Victor Babes” University of Medicine and Pharmacy, E. Murgu Sq., No. 2, 300041 Timisoara, Romania; 7 Faculty of Food Science and Technology, University of Agricultural Science and Veterinary Medicine, 400372 Cluj-Napoca, Romania; zorita.sconta@usamvcluj.ro; 8 National Institute of Research and Development for Electrochemistry and Condensed Matter, 144 Dr. A. P. Podeanu, 300569 Timisoara, Romania; adina.cata@yahoo.com; 9 Romanian Academy “Coriolan Dragulescu”, Institute of Chemistry, Bv. M. Viteazu, No. 24, 300223 Timisoara, Romania; danielahaidu1@gmail.com; 10 Faculty of General Medicine, “Carol Davila” University of Medicine and Pharmacy, Dionisie Lupu Street, No. 37, Sector 2, 020021 Bucharest, Romania; andreea.paunescu@stud.umfcd.ro

**Keywords:** *Aronia melanocarpa* (Michx.) Elliott, heavy metals, phytochemical composition, antiproliferative, cell viability, apoptosis, necrosis, rutin

## Abstract

Black chokeberry (BCK), known as *Aronia melanocarpa* (Michx.) Elliott, has been employed for various purposes throughout history, being exploited both for its nutritional properties (functional foods, beverages, food preservatives, and natural food colorants) and for its therapeutic benefits (including cardiovascular and metabolic settings). This paper presents the first report on the identification of inorganic elements in three standardized BCK extracts: frozen berries (FrozArs), dried berries (DryArs), and evaporated juice (EvArJ). Additionally, the antiproliferative and pro-apoptotic effects of these extracts on human pulmonary artery endothelial cells (HPAECs) were evaluated. Concentrations ranging from 1 μg/mL to 10 μg/mL were tested. Inorganic element analysis revealed detectable levels of metals, including aluminum (Al), cadmium (Cd), chromium (Cr), copper (Cu), iron (Fe), manganese (Mn), and zinc (Zn). Notably, cadmium was found in very low amounts (0.026 μg/g in the FrozArs), while iron was the most abundant element in the juice (597.665 μg/g). MTT assays demonstrated that all three extracts exhibited antiproliferative activity against HPAECs. Cell cycle analysis revealed a decrease in the G2/M phase for all extracts, along with an appearance of the sub-G0 phase at the highest concentration tested. The DryAr extract also slightly reduced the number of cells in the G0-G1 phase. Annexin V/PI staining indicated a mild increase in the percentage of necrotic cells associated with the DryAr extract. The potential implications of these findings are significant, particularly for those interested in the health effects of dietary supplements.

## 1. Introduction

Cardiovascular disease (CVD) is the leading cause of death worldwide, causing about one in three deaths globally and more than half of the deaths in Romania [1,2,3]. These statistics are concerning, as nearly half of Romanian deaths are linked to behavioral risk factors like poor diet, physical inactivity, tobacco use, and alcohol consumption [3]. The vascular endothelium, a thin layer of cells lining blood vessels, is vital for regulating blood pressure and maintaining vascular health [4]. Early aging of the endothelium due to cardiovascular risk factors can lead to hypertension and other CVDs [5,6]. Endothelial dysfunction (ED) is a condition characterized by the loss of nitric oxide (NO) availability and inflammatory processes in the endothelium, followed by vasoconstriction and a pro-thrombotic state. Moreover, high levels of oxidative stress (OS) contribute to vessel damage and further aggravate ED, causing hypertension and CVDs [7,8]. ED, hypertension, and CVDs are all connected to genetic and behavioral risk factors, such as sedentarism, smoking, and poor dietary habits [5,8,9]. Technological progress and modernization have caused a shift in our lifestyles towards a more desk-bound lifestyle and unhealthy dietary patterns consisting of processed foods, rich in salt, animal fats, and refined sugars, and low in fiber, vegetables, and fruit, negatively impacting overall health [10,11]. This shift negatively impacts overall health [10,11]. In the long run, these behavioral patterns can lead to low-grade chronic inflammation, weight gain, increased waist circumference, dyslipidemias, decreased high-density lipoprotein cholesterol (HDL-cholesterol), increased insulin resistance, and high glucose levels, all of which contribute to metabolic dysregulation. This metabolic dysregulation, along with other factors, can cause the so-called “modern-life diseases” (i.e., obesity, atherosclerosis, hypertension and other CVDs, and type 2 diabetes mellitus) [10,12].

In light of this, addressing modifiable risk factors is the first recommendation for the prevention and management of CVDs and metabolic dysfunction before considering pharmacological interventions [13]. Firstly, engaging in regular physical activity promotes weight loss, lowers blood pressure, and enhances both cardiovascular and muscular endurance. This, in turn, reduces the risk of cardiovascular and metabolic diseases [11,14]. Secondly, adopting a healthy diet that includes lean proteins, whole grains, fruits, and vegetables offers numerous health benefits. Such a diet can prevent constipation, lower cholesterol and blood sugar levels, and reduce low-grade chronic inflammation, all due to its high content of dietary fiber, micronutrients (e.g., vitamins and minerals), and phytochemicals (e.g., phytoantioxidants) [15,16]. Lastly, there is growing interest in turning to phytotherapy because herbal remedies are perceived as safer and more cost-effective than conventional medicines, with them also having other nutritional benefits for the human body [13,14,17].

Berries are gaining well-deserved recognition among nutritionists worldwide because they are rich in antioxidants, minerals, and vitamins while being low in calories [18]. The vibrant blue, purple, and red colors of these fruits are a result of their high contents of anthocyanins, a class of polyphenols that have been extensively studied for their potential uses in nutraceuticals [19,20,21,22]. Consuming foods rich in anthocyanins should be prioritized, as research suggests that it is associated with a lower incidence and progression of chronic disorders characterized by chronic low-grade inflammation and elevated levels of reactive oxygen species (ROS). These disorders include CVDs [19,23], diabetes [19], obesity [24], and neurodegenerative conditions [20,21].

*Aronia melanocarpa* (Michx.) Elliott, commonly referred to as black chokeberry (BCK), is a cultivated shrub belonging to the *Rosaceae* family and the Amygdaloidae subfamily. This species originates from wild populations found in the eastern regions of North America [8,25,26]. Although its fruits are not usually eaten fresh due to their astringent flavor, they are highly regarded for their numerous health benefits. They are commonly used in various products, including beverages (such as juices, wine, liqueurs, and fruit teas), jams, syrups, dietary supplements, and as a natural food dye [8,25]. The astringent taste of BCKs is primarily due to their high concentration of polyphenols and tannins. BCK is recognized as one of the most significant sources of phenolic bioactive compounds, including proanthocyanidins, anthocyanins, flavonols, and phenolic acids [27,28,29]. Specifically, anthocyanins and proanthocyanidins—such as cyanidin-3-*O*-glucoside, cyanidin-3-*O*-galactoside, and cyanidin-3-*O*-arabinoside—are mainly responsible for the fruit’s antioxidant properties [30,31]. In addition to these compounds, BCKs contain a variety of organic acids (including chlorogenic acid, neochlorogenic acid, benzoic acid, caffeic acid, malic acid, and citric acid), vitamins (such as B2, B3, B9, C, K, carotenoids, and tocopherols), and minerals (including sodium, potassium, calcium, magnesium, iron, zinc, phosphorus, and chromium) [26,27,28,29,32]. Furthermore, BCKs are nutritious, offering significant amounts of dietary fiber, carbohydrates (like fructose, glucose, and sorbitol), and amino acids (including arginine, cysteine, lysine, and tyrosine), while containing relatively low levels of fats (such as polyunsaturated fatty acids, linoleic acid, oleic acid, sterols, and phospholipids) [26,27,28,29,32]. The chemical composition of BCKs can vary based on local conditions (such as soil type and climate), harvest timing, and storage methods [26].

The medical benefits of this vegetal product administrated in various forms include antioxidant properties, support for cardiovascular health, anti-inflammatory effects, anti-diabetic effects, anticancer potential, boosts to the immune system, and neuroprotective effects [26,28,30].

The present study aimed to (a) perform an analysis of inorganic elements to determine the presence or absence of heavy metals in three types of black chokeberry (BCK) extracts, specifically those obtained from frozen berries (FrozArs), dried berries (DryArs), and evaporated juice (EvArJ), and (b) evaluate the antiproliferative and proapoptotic effects of these extracts on a human pulmonary artery endothelial cell (HPAEC) line, as recent scientific studies have highlighted the potential of BCK extracts in managing cardiovascular and metabolic disorders, with most published data being focused on animal models or human subjects [33].

All these comprehensive steps have been taken to ensure the reliability of the data for a subsequent clinical trial aimed at assessing the potential significant cardio-metabolic effects of organic BCK preparations sourced from a plantation in the western part of Romania.

## 2. Results

### 2.1. HPLC

The liquid chromatography analysis coupled with diode array detection and electrospray ionization tandem mass spectrometry, as shown in Table 1, identified 10 polyphenolic compounds, including those from the anthocyanin and flavonol classes, as well as phenolic compounds such as hydroxycinnamic acids. As already reported in our previous article, the dry fruit extract has flavonols (i.e., rutin, 6136.61 μg/g), followed by hydroxycinnamic acids (i.e., caffeic acid, 4224.30 μg/g, chlorogenic acid, 3024.26 μg/g, and neochlorogenic acid, 1394.38 μg/g) and flavonols (i.e., quercetin-3-O-glucoside, 2985.63 μg/g), as its main components [34]. Secondly, the frozen fruit extract contains mainly anthocyanins (i.e., cyanidin-3-O-glucoside, 7562.22 μg/g), flavonols (i.e., rutin, 5989.25 μg/g), and hydroxycinnamic acids (i.e., caffeic acid, 3752.98 μg/g and chlorogenic acid, 3024.26 μg/g) [34]. Herein, we report, for the first time, the phytochemical composition of the evaporated organic juice extract, which contains hydroxycinnamic acids (i.e., neochlorogenic acid, 433.45 μg/g, and chlorogenic acid, 384.31 μg/g), anthocyanins (i.e., cyanidin-3-*O*-glucoside, 67.92 μg/g), and flavonols (i.e., quercetin-3-*O*-glucoside, 53.34 μg/g) as its major components.

### 2.2. Inorganic Element Identification

Aiming to certify the safety of their administration, the presence of inorganic elements was evaluated in the three extracts. The obtained results are presented in Table 2.

As shown in Table 2, all three extracts contain detectable values of aluminum (Al), cadmium (Cd), chromium (Cr), copper (Cu), iron (Fe), manganese (Mn), and zinc (Zn). The results highlighted a minimum concentration of Cd in the frozen fruit extract of 0.026 µg/g and a maximum concentration of Fe in the juice of 597.665 µg/g. More precisely, iron (Fe) was the most quantitatively significant metal detected, with concentrations of 302.667 µg/g in the frozen fruit extract, 396.728 µg/g in the dried fruit extract, and 597.665 µg/g in the juice extract. In comparison, the cadmium (Cd) concentrations were 0.026 µg/g in the frozen fruit extract, 0.043 µg/g in the juice extract, and 0.049 µg/g in the dried fruit extract.

Furthermore, besides Fe, aluminum (Al) and zinc (Zn) were the most abundant elements in the analyzed extracts. The aluminum levels were 277.355 µg/g in the dried fruit extract, 329.037 µg/g in the frozen fruit extract, and 380.039 µg/g in the juice extract. The zinc concentrations were measured at 44.344 µg/g in the dried fruit extract, 58.909 µg/g in the frozen fruit extract, and 44.708 µg/g in the juice extract.

Among the elements found to a lesser extent, we can mention chromium, whose concentrations in the extracts were 0.026 µg/g in the frozen fruit extract, 0.043 µg/g in the juice extract, and 0.049 µg/g in the dried fruit extract. Also, copper was quantified at 0.243 µg/g in the juice extract, 0.282 µg/g in the frozen fruit extract, and 0.343 µg/g in the dried fruit extract. Manganese was present at 1.418 µg/g in the dried fruit extract, 1.177 µg/g in the frozen fruit extract, and 1.550 µg/g in the juice extract.

Heavy metals, such as arsenic (As), cobalt (Co), nickel (Ni), and lead (Pb), were determined to have concentrations below the limit of detection.

### 2.3. In Vitro Analysis

#### 2.3.1. MTT Assay

**Figure 1:**
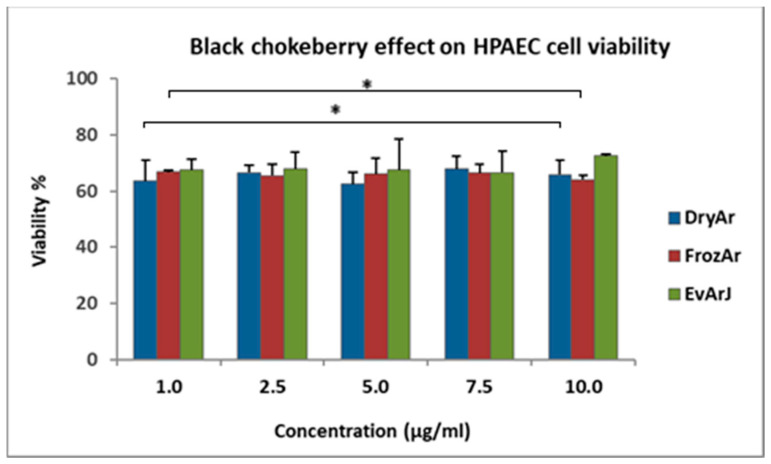
Results of the in vitro antiproliferative analysis of the BCK extracts on the HPAECs after 72 h of exposure to the selected samples using the MTT method (Data = mean ± SEM, * *p* < 0.05, vs. control [one-way ANOVA]). Legend: DryArs—extract obtained from dried berries; FrozArs—extract obtained from frozen berries; EvArJ—extract obtained from evaporated BCK juice.

Figure 1 presents the results for the antiproliferative effects of the BCK extracts on the HPAEC line, and Table 3 provides the numerical data for the in vitro antiproliferative assessment.

Analyzing the MTT results, one can observe that all three extracts exerted a similar antiproliferative effect on the human pulmonary artery endothelial cell (HPAEC) line without a dose-dependent relationship in the studied concentration range (1–10 μg/mL). After exposing the cells for 72 h to the tested extracts, the cell viability was as follows: for the DryArs, the viability ranged between 62.58 ± 4.28% and 67.85 ± 4.57%; for the FrozArs, the viability ranged between 64.17 ± 1.46% and 66.89 ± 0.63%; and for the EvArJ, the viability ranged between 66.62% ± 7.54% and 72.57 ± 0.76%. In the case of the EvArJ, a slight tendency for cell viability to increase was observed after exposure to the 10 μg/mL concentration, but this was statistically insignificant (*p* > 0.05). These results were compared to those for untreated (control) cells, whose viability was 100%. The highest DMSO solvent concentration (0.01%) in the analyzed samples did not significantly influence cell viability.

#### 2.3.2. Cell Cycle Analysis

**Figure 2:**
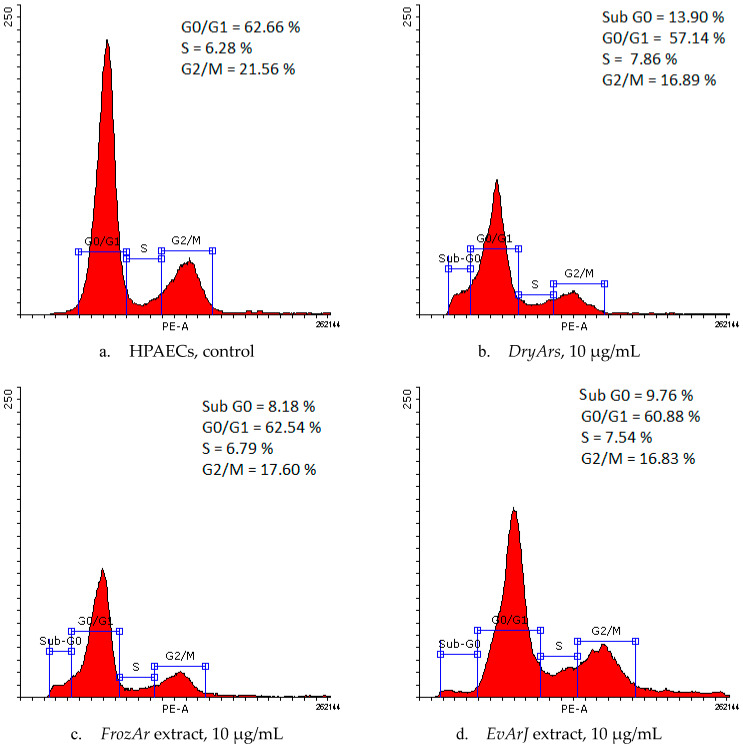
Histograms of HPAEC cycle phases: control (**a**) and after exposure for 72 h to BCK extracts, 10 μg/mL (**b**–**d**).

The evaluation of the results of the cell cycle analysis, provided by flow cytometry (Table 4 and Figure 2), reveals slight changes in the cell cycle phases after the cells were exposed to the BCK extracts. Decreases in the percentage of cells in the G2/M phase (from 21.56 ± 2.23% (control) to 16.83 ± 3.35% (EvArJ, 10 μg/mL), 16.85 ± 5.42% (DryArs, 10 μg/mL), and 17.60 ± 4.31% (FrozArs, 10 μg/mL) were observed, with the appearance of a sub-G0 phase for the highest concentration of the three BCK extracts: 13.90 ± 8.67% (DryArs), 8.18 ± 3.86% (FrozArs), and 9.76 ± 3.99% (EvArJ). For the DryArs, a decrease in the G0-G1 phase percentage (57.14 ± 4.30%) at 10 μg/mL was observed compared to the control, where the percentage of the G0-G1 population was 62.66 ± 4.22%.

#### 2.3.3. Annexin V/PI Analysis

**Figure 3:**
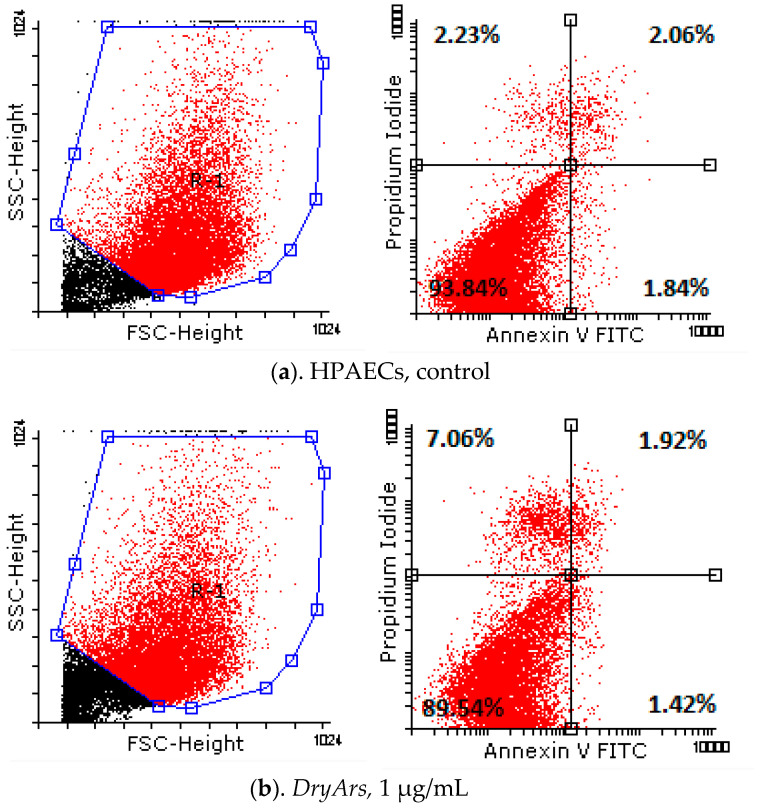

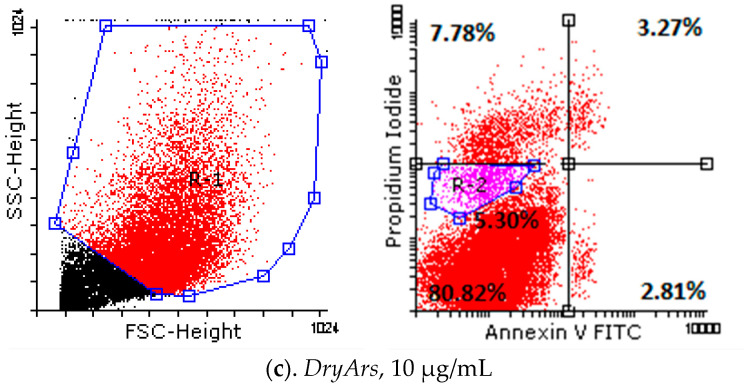
Annexin V/PI analysis diagrams of HPAECs after exposure for 72 h to tested BCK extracts.

Analyzing the obtained results (Table 5, Figure 3) from testing the cell death of the HPAECs after exposure for 72 h to the BCK extracts using the Annexin V/PI method, a decrease in living cells from 93.84 ± 0.77% (control cells, untreated) to 89.54 ± 2.27% in the case of exposure to the DryAr extract (1 μg/mL), accompanied by a slight increase in the percentage of necrotic cells, from 2.23 ± 0.14% in the case of the control to 7.06 ± 2.99% in the case of exposure to the DryAr extract, was observed. As the extract concentration increased, the percentage of living cells decreased to 80.82 ± 4.86% (DryAr extract, 10 μg/mL), and a new population of dying cells, R2, emerged at 5.30 ± 1.21%, with the percentage of necrotic cells being 7.78 ± 4.20%. In the case of exposure of the HPAECs to the FrozAr extract, a very slight increase in the rate of cells in the early apoptosis and late apoptotic phases, independent of the concentration to which the cells had been exposed, was noted without statistical significance. Notably, no changes were observed for the BCK evaporated juice extract.

## 3. Discussion

Romania has one of the lowest life expectancies in the European Union (EU), with a significant number of deaths directly attributed to CVDs [35]. The situation is challenging, as the mortality rate from treatable causes is more than double the EU average, and mortality from preventable causes is the third highest in the EU. Moreover, three-quarters of Romanian adults reported not eating vegetables or at least one fruit per day [36]. These alarming statistics highlight the immediate need for strategies to address cardiovascular risk factors, including behavioral issues, such as unhealthy diets [35,36].

In light of this, as we plan to conduct a further clinical trial assessing the cardio-metabolic effects of organic Romanian BCK preparations, firstly, we aimed to comprehensively characterize the phytochemical composition of three BCK extracts (obtained from dried and frozen berries and evaporated juice) to evaluate their inorganic composition and to test their antiproliferative/proapoptotic effects in a cell line model of endothelial cells (used to study cardiovascular diseases/vascular homeostasis), in order to ensure their safety.

### 3.1. Phytochemical Composition

As presented in Table 1 and [34], the chromatographic analysis of the extracts revealed that the extracts’ chemical composition depends on the way the extracts are processed. More precisely, the extract derived from the frozen berries is particularly rich in anthocyanins (e.g., Cy-3-*O*-glucoside), flavonols (e.g., rutin), and hydroxycinnamic acids (e.g., caffeic and chlorogenic acid), resulting in the highest concentrations of active substances in terms of polyphenols and simple phenols (total phenolic content). The extract from the dried berries is high in flavonols (e.g., rutin), hydroxycinnamic acids (e.g., caffeic and chlorogenic acid), and other flavonols (Q-3-*O*-glucoside), ranking second for its polyphenol and simple phenol content. In contrast, the evaporated juice extract shows the lowest concentrations of total phenolic compounds. However, it still contains some phenolic compounds, such as hydroxycinnamic acids (neochlorogenic acid and chlorogenic acid) and polyphenols, including anthocyanins (Cy-3-*O*-glucoside) and flavonols (Q-3-*O*-glucoside). The extracts that were chosen for testing fall within the limits reported in the literature, according to their phytochemical content [37,38,39]. Unsurprisingly, it was found that the extract obtained from evaporated juice had the lowest concentration of polyphenols and simple phenols (overall, in terms of total phenols) (1042.93 μg/g), almost 20 times lower than that of the dry fruit extract, with the main components being simple phenols and, to a much lesser extent, polyphenols. Sidor and Michalowska also assessed the qualitative composition and phenolic load of BCKs and found that the main components in the juice are phenolic compounds, i.e., hydroxycinnamic acids [29]. This can be explained by (a) the processes that the BCKs undergo during juice preparation, which may decrease the concentration of anthocyanins, (b) the conditions in which the juice is stored after its preparation, and (c) the storage time, which can negatively impact the concentration of anthocyanins and, thus, of polyphenols [40,41].

On the other hand, the frozen BCK extract exhibited the highest concentration of total phenolic compounds, significantly higher than both the dry fruit extract and the juice. Salazar-Orbea et al. studied the influence of the preparation technique and storage time on the polyphenolic content in strawberries and apples, and they also found that freezing preserves the polyphenolic content in the fruit best. This is because the freezing of the fruit decreases and inhibits the activity of the enzymes responsible for polyphenol oxidation and degradation [42]. Anthocyanins were best preserved in the FrozAr extract, suggesting that this processing technique should be preferred to juicing to better reap BCKs’ antioxidant benefits. Further studies are required to evaluate and establish the most appropriate pharmaceutical form of BCK extract in terms of the bioavailability and stability of its bioactive compounds.

### 3.2. Inorganic Element Determination

In light of the widespread use of BCK due to its numerous beneficial effects on human health and considering that certain metals can be detected in this plant species, it is necessary to investigate both the plant species and the products obtained from it for the presence of harmful elements [26,43]. Thus, identifying highly toxic metals was paramount, mainly because scant research has been conducted on the mineral composition of black chokeberries, aiming to certify their safety.

The analysis of the BCK extracts confirmed the presence of essential and trace metals (Table 2), including Al, Cd, Cr, Cu, Fe, Mn, and Zn, with varying concentrations across the different extract types. As aforementioned, the lowest detected concentration was observed for Cd in the frozen fruit extract, while the highest was Fe in the juice extract, and these results are consistent with some of the literature data [44,45]. Moreover, similar determinations were obtained by Kaličanin et al. in *Aronia* berries using two different analytic methods: 0.043 µg/g (potentiometric stripping analysis, PSA) and 0.049 µg/g (inductively coupled plasma optical emission spectroscopy technique, ICP-OES) [45,46]. For juice, they obtained about 10-fold lower Cd values (0.0042–0.0043 µg/g), which could be explained by the fact that, in our study, the alcoholic extract from fruit and juice was tested, which was concentrated until total solvent removal [43]. It is worth noting that Mężyńska et al. conducted several in vivo studies reflecting the environmental exposure of humans at low-level and moderate Cd concentrations, and they demonstrated that the berry extract from *A. melanocarpa* L. offers protection from the development of oxidative stress in the liver and improves this organ’s morphology in rats [46].

Our results indicate that certain metals, notably Fe, Al, and Zn, were present in the extracts at concentrations exceeding those reported in previous studies. In particular, iron (the most abundant metal detected) was quantified at 302.667 µg/g in the frozen fruit extract, 396.728 µg/g in the dried fruit extract, and 597.665 µg/g in the juice extract. These values are substantially higher than those documented in the literature, such as the ranges reported by Jurendić (9–25 µg/g in Aronia juice and fruits), Pavlović (9.4–58.1 µg/g in tea, Aronia juice, and fruits), and Pieszka (197 µg/g). Although aluminum was detected at higher levels than other metals, the concentrations remain within the safe limits established by regulatory guidelines and do not pose a health risk [47,48,49,50]. It is important to note that, although the estimated aluminum intake remains within the EFSA-established TWI for an average adult, certain populations—such as children, the elderly, or individuals with impaired renal function—may be more vulnerable to aluminum accumulation. Therefore, long-term exposure and total dietary intake should be considered when evaluating safety in these groups [51,52,53]. In some cases, zinc concentrations are 10 times higher than those reported in some studies but are not significant enough to reach the recommended daily intake [26].

The concentrations of metals such as cadmium (Cd), copper (Cu), and manganese (Mn) in the analyzed samples were lower than those reported in the existing literature. Nevertheless, they remain relevant for mineral supply from natural sources [44,50,54,55]. The levels of copper detected suggest that BCK extracts contribute minimally to dietary copper intake, remaining within safe and acceptable limits. These concentrations are lower than those reported by other authors, including 0.8–2 µg/g (berries), 1–5 µg/g (juice), and 5–12 µg/g (pomace) [26], as well as various contents ranging from 0.82 to 4.51 µg/g (for tea, juice, and berries) [49,50]. Additionally, the manganese levels indicate that chokeberry extracts contribute only modestly to manganese intake, posing no risk of excessive accumulation, especially since they are low compared to those identified in another study (5.49–17.89 µg/g) [49]. The detected chromium (Cr) levels were within the expected ranges for plant-based products and did not exceed toxicological safety limits. Similar chromium concentrations have been reported in the literature for Aronia berries at 0.049–0.053 µg/g, as determined by the ICP-OES method [49], and at 0.05 µg/g and 0.06 µg/g in juice [26].

The variations in metal content in BCK extracts are influenced by a multitude of factors. These include soil composition, the maturation state of plants, climate conditions, and genetic differences. These factors collectively affect the uptake of elements. Notably, the observed differences may be linked to the acidity of the chokeberry fruit, as metals tend to accumulate more readily in acidic environments. The characteristics of the plants’ cultivated soil and the capacity of polyphenols to chelate metals are also contributing factors. Additionally, differences in methodological approaches across studies may offer an additional explanation for the observed discrepancies in the results [49,50].

The concentrations of arsenic, cobalt, nickel, and lead were determined to be below the detection limit, reaffirming the safety of BCK extracts. According to the European Medicines Agency (EMA), the permitted concentration of class 1 elemental impurities like Cd, Pb, and As is 0.5 µg/g (Cd), 0.5 µg/g (Pb), and 1.5 µg/g (As) [56]. Also, these findings comply with the World Health Organization (WHO) guidelines for evaluating the quality of herbal medicines regarding contaminants in finished dietary supplements, which set maximum limits of 0.01 mg/day for As, 0.02 mg/day for Pb, 0.006 mg/day for cadmium (Cd), and 0.02 mg/day for chromium (Cr) [57]. Moreover, this study’s findings support the potential of BCK extracts as a nutrient-rich dietary component with multiple health benefits. Hence, the absence of toxic heavy metals in significant concentrations, along with the presence of essential trace elements, reinforces the safety and nutritional value of BCK-based products, offering a promising avenue for further research and potential health applications [26,29,44,54,56].

### 3.3. In Vitro Evaluation of the Selected Samples

The in vitro evaluation of the selected samples demonstrated that DryAr, FrozAr, and EvArJ extracts impact HPAEC proliferation even at low concentrations (1–10 µg/mL). Although a reduction in cell proliferation was observed across all tested extracts, no IC50 (half-maximal inhibitory concentration) value could be determined within the investigated dose range, and no clear dose-dependent effect was identified by the MTT cell proliferation assay.

Cell cycle analysis (Table 4) further corroborated the MTT results (Table 3), indicating a consistent trend across all extracts. A slight dose-dependent increase in the subG0 phase population was observed, suggesting potential cellular stress or apoptotic induction, with the DryAr extract exhibiting the most pronounced effect. Additionally, Annexin V/PI staining confirmed a slight decrease in the proportion of viable cells and a concomitant increase in necrotic cells for the DryArs, while no such trend was observed for the FrozArs and the EvArJ (variation in rutin concentrations among the tested extracts: 31.92% in the DryArs, 22.42% in the FrozArs, and 4% in the EvArJ, compared with the total phenol content).

Several research groups have conducted extensive studies on the antiproliferative and cytotoxic effects of BCKs, particularly focusing on cancer cell lines compared (or not) to normal cell lines. The results of these studies vary significantly based on several factors, including the type of extract used, the specific part of the plant used, its origin (such as latitude and soil composition), the time of harvest, and the type of cancer/non-cancer cell line examined. It is well documented in the literature that there is often a notable difference in the antiproliferative effects observed in cancer cell lines compared to normal cell lines. In the following sections, the most significant studies that have compared the antiproliferative effects of black chokeberry extracts on cancer cell lines versus normal cell lines will be highlighted.

The study performed by Malik M et al., which assessed the cytotoxicity (flow cytometry method) of BCK extracts on normal colon cells (NCM460) and cancer cells (human HT-29 colon cancer cells), reported no cytotoxic effects on normal colon cells, although anticancer activity was observed on the cancerous line. After 24h of exposure at 50 mcg (ethanolic extract), the semipurified anthocyanin-rich BCK extract induced a 60% growth inhibition on the treated cancer line. The cell cycle’s G1/G0 and G2/M phases were also blocked/affected, as reported by the present study. This further induced an increased expression of p21WAF1 (cyclin-dependent kinase inhibitor 1) and p27KIP1 (multifunctional cyclin-dependent kinase inhibitor with prognostic significance in human cancers) genes and decreased cyclin A and B gene expression. Moreover, no further effects were observed after the prolonged exposure of cancer cells to the extract (in terms of cell number), thus suggesting a cytostatic effect [57]. The normal colon cells expressed minimal growth inhibition (<10%) at the maximum concentration used (i.e., 50 mcg/mL) [57].

In another study, Gill NK et al., 2021, tested three commercially available extracts of three types of Aronia berries, red, purple, and black, for their phytochemical composition (total phenols) and antioxidant and inhibitory activity against the same cancer cell line (HT-29 human colon cancer cells). The BCK extract was the only one presenting anticancer activity (MTT assay), and its activity correlated/was in line with its phenolic content (85.2 mg gallic acid equivalent, GAE/g extract), antioxidant activity, and caffeic and chlorogenic acid levels [58].

A recent study, published in 2024 and performed by Dvorska D et al., investigated the effects of BCK extract (a fruit peel methanolic extract rich in anthocyanins and procyanidins) on both breast cancer cell lines (MCF-7 and MDA-MB-231) and normal cell lines (MCF-10A and BJ-5ta). The results showed that the BCK extract induced a concentration-dependent inhibition of metabolic activity both in cancer and normal cell lines, observed even at lower doses [59]. This study supports our recent finding regarding BCKs’ antiproliferative potential in normal cell lines.

The study performed by Bermudez-Soto MJ and colleagues in 2007 on the Caco-2 cell line of human colon cancer highlighted that polyphenol-rich BCK juice inhibited the G2/M phase, an aspect that correlates with our findings. The authors reported that cell proliferation was inhibited in the G2/M phase, and the viability of cells decreased for all the tested extracts [60].

Also, the study performed by Sharif et al. in 2013 confirmed our findings and showed that BCK juice (7.15 g/L) rich in polyphenols induced an antiproliferative effect on the acute lymphoblastic leukemia Jurkat cell line and provoked a G2/M phase cell cycle arrest, which caused the induction of apoptosis [61].

In 2024, the research group led by Nowak D evaluated the anticancer properties of two types of juice: *Aronia melanocarpa* and *Morinda citrifolia*. Tests were carried out on a urinary bladder cancer cell line (T24) as well as a normal uroepithelial cell line (SV-HUC1). Both juices affected cell viability in a time- and concentration-dependent manner, showing greater cytotoxicity in the cancer cell line. Notably, the BCK juice was found to be more effective at lower concentrations (1.56%) compared to Noni juice (12.5%). This difference is likely due to the BCK juice’s higher antioxidant capacity, attributed to its greater polyphenol content. Additionally, higher doses of the BCK juice decreased cell viability in both normal and cancer cell lines. At a concentration of 1.56%, the cell viability for normal cells was around 90%, whereas at 3.125%, it dropped below 50%. However, the impact on cancer cells was more significant. Results from the MTT assay, which calculated lethal concentration values (LCs), defined as the percentage of cell death, revealed that higher concentrations of the BCK juice (1.56% and 3.125%) resulted in much higher LC50 and LC90 values for normal cells compared to cancer cells. Specifically, the LC50 values were 3.04 for normal cells versus 2.17 for cancer cells, and the LC90 values were 9.56 for normal cells versus 7.02 for cancer cells, with these differences being statistically significant. It is important to note that BCK juice is rich in phenolic acids (such as caffeic and chlorogenic acids), flavonols (like quercetin), and flavanols (such as epicatechin) [62].

Borczak et al. investigated the antiproliferative effects of four types of wild-growing fruit—chokeberry, elderberry, hawthorn, and sea-buckthorn—on normal BJ fibroblast lines, the MCF-7 breast cancer cell line, and WM793, a melanoma cell line. They used methanol–acetone extracts at concentrations ranging from 0.5 mg/mL to 2.5 mg/mL. For the BCK extract, a non-concentration-dependent effect on cell viability was observed in the normal cell line: at 0.5 mg/mL, cell viability was 81.05%; at 1.0 mg/mL, it was 87.18%; at 1.5 mg/mL, it was 83.64%; and at 2.5 mg/mL, it was 85.08%. In contrast, other extracts, particularly the elderberry extract, demonstrated a concentration-dependent effect in decreasing cell viability. Additionally, the effects of BCK exposure varied significantly among the cell lines. At a concentration of 0.5 mg/mL, cell viability after BCK exposure was 81.05% for the normal fibroblast line, 96.78% for the melanoma cell line, and only 39.74% for the breast cancer cell line. At a concentration of 2.5 mg/mL, the cell viability measurements were 85.08% for fibroblasts, 89.37% for melanoma cells, and 37.63% for breast cancer cells. These results suggest that BCK extract has differing effects on pathological and non-pathological cell lines [63].

Regarding rutin, a flavonoid that was found to be more abundant in the DryAr extract, its cytotoxic effect has been reported by some studies in the literature. Therefore, it is worth mentioning the study performed by Bushmeleva K’s group and published in 2023 regarding BCK’s antiradical and immunomodulating activities. This study involved isolating flavonol compounds from a fruit extract and evaluating their activities, including cytotoxicity, on the lymphoblast cell line RMPI-1788. The results indicated that at lower concentrations, the flavonol fraction had a positive impact on normal cell lines, while at higher concentrations, it induced cytotoxic effects [64]. This finding aligns with previous research by Matsuo M et al., which demonstrated the cytotoxicity of nine flavonoids on normal cultured cells, including TIG-1 (human lung embryonic fibroblasts) and HUVECs (human umbilical vein endothelial cells). Matsuo M concluded that certain flavonoids showed significant cytotoxicity at relatively low concentrations in a dose-dependent manner, with the degree of cytotoxicity varying based on the specific molecule, concentration, and cell line tested. Interestingly, rutin was found to be nontoxic to both tested cell lines. The authors of the study suggested that this non-cytotoxicity may be attributed to rutin’s higher hydrophilicity, which limits its incorporation into cells. Additionally, they concluded that some cancerous cell lines may exhibit greater sensitivity to flavonols than others [65].

In conclusion, based on the previously mentioned aspects regarding the cytotoxicity of flavonoids and other related research findings, it can be stated that at high concentrations, flavonoids can increase the intracellular levels of reactive oxygen species (ROS). Moreover, the effects induced by different combinations of flavonoids, whether in an extract or a mixture, are not only diverse but also fascinating. These effects can be agonistic, synergistic, antagonistic, or a combination of these, depending on the method used to evaluate the antiradical activity of the tested samples [66,67]. Additionally, in a paper published in 2022, Joshi T concluded that rutin hydrate (a glycoside of quercetin) exhibited less antioxidant activity compared to quercetin dihydrate, its aglycone counterpart. Generally, aglycones tend to have greater antioxidant potential than their glycoside forms; however, glycosides, like rutin, can enhance bioavailability [68,69]. Nevertheless, in this study, the cellular stress induced by the DryAr extract cannot be attributed solely to rutin but rather to the synergistic action of all the active compounds present in the extract.

The 2018 study conducted by Cvetanovic A et al. investigated three types of BCK extracts—derived from stems, leaves, and berries—and their effects on three malignant cell lines: A-549 (human lung adenocarcinoma), LS-174T (human colorectal adenocarcinoma), and HeLa (human cervical adenocarcinoma). The researchers compared these effects to those on the MRC-5 cell line, which consists of normal lung fibroblasts. The findings indicated that the HeLa cell line is particularly sensitive to BCK extracts, especially those obtained from the leaves. The IC50 values for the normal cell line were as follows: 5.50 mcg/mL for the berries and 1.72 mcg/mL for the leaves. Interestingly, these values were similar to those observed for the malignant LS-174T cell line. The observed differences in the activity of the extracts were linked to the significantly higher total phenolic content in the leaves, which was ten times greater than that in the berries. This suggests a synergistic effect among the different constituents of the extracts. Furthermore, the study found a correlation between the cytotoxic activity of the extracts, their antioxidant capacity (as measured by the DPPH assay), and their phytochemical content. This provides a scientific basis for the effects observed in the study. Rutin was identified as the predominant compound present in all extracts, with the highest concentration found in the berries [66].

A study published by Caparica R et al. in 2020 examined the flavonol rutin and its effects on cell viability. The researchers tested various concentrations of rutin (ranging from 0 to 250 µM) on non-cancerous Vero kidney cells using an MTT assay over 48 h. They found that higher concentrations of rutin (100 µM and 250 µM) led to a significant decrease in cell viability, specifically 65.6% and 52.1%, respectively. In contrast, the impact of rutin on 786-O cancer cells (a human renal cancer cell line) resulted in a notable, concentration-dependent reduction in cell viability, even at lower concentrations. For these cancer cells, the viability decreased by 56.1% at 50 µM, 32.4% at 100 µM, and 25.3% at 250 µM. The authors concluded that rutin did not exhibit significant cytotoxicity at concentrations of up to 50 µM, but higher concentrations caused notable decreases in viability in normal cells. Therefore, to ensure both efficacy and safety, the researchers recommend that rutin should be used at a maximum concentration of 50 µM in future studies. Additionally, the study indicated that rutin induced an increase in the sub-G1 population (approximately 30%) and an increase in the S-phase population, along with a subsequent decrease in the G0/G1 population of the cancer cell line [67].

The studies conducted by Mladenka P. et al. (2009) and Filipsky T. et al. (2017) concluded that rutin can induce pro-oxidant effects and exacerbate cardiotoxicity, depending on the dosage [70,71].

However, as already mentioned, the differences observed for the DryAr extract in terms of necrosis induction, compared with other extracts, cannot be attributed only to rutin in particular, as our molar concentration for rutin in the 10 μg/mL extract solution was 0.10 μM, which is extremely low compared to the abovementioned reported data.

Research on the effects of BCKs on cardiovascular-related cell lines is limited and requires further investigation. Most studies have focused on animal models and human subjects, with few data available from cell line experiments.

Borechi K et al., in 2016, conducted a study on the effects of a polyphenol-rich BCK extract (commercially available) on tumor necrosis factor-alpha (TNF-α)-induced apoptosis in a cell line of cardiomyoblasts (H9c2). The study found that at 50 mcg/mL, the extract induced the strongest apoptosis and cytotoxicity (IC50: 55.84 mcg/mL), suggesting a potential cardioprotective action of BCKs on cardiac cells exposed to inflammatory conditions [72].

Several studies evaluated BCKs’ effect on vascular endothelial cells. The study conducted by Zapolska-Downar D et al., 2020, on human aortic endothelial cells (HAECs) concluded that BCK extract significantly inhibited ICAM-1 (intercellular adhesion molecule-1) and VCAM-1 (vascular cell adhesion molecule-1), attenuated the phosphorylation of NF-kBp65, and reduced ROS production in TNF-α treated HAECs, thus suggesting anti-inflammatory effects, through the modulation of adhesion molecules and oxidative stress reduction, in vascular endothelial cells [73].

Three studies on the human umbilical vein endothelial cell (HUVEC) line regarding BCK extracts’ effects have been published. Luzak et al., in 2010, showed the concentration-dependent antiplatelet effect of a BCK extract on HUVECs [74]. Iwashima T et al., 2019, showed that an anthocyanin-rich BCK extract managed to decrease TNF-α inflammation and to decrease the mRNA (messenger ribonucleic acid) levels of several pro-inflammatory markers (i.e., interleukins (IL)-1β, IL-6, and IL-8) and MCP-1 (monocyte chemoattractant protein-1). Moreover, it decreased the adhesion of monocytes to endothelial cells by decreasing VCAM-1 expression [75]. Zielinska et al., in their study published in 2024, evaluated the anti-atherosclerotic properties of BCKs. The extract inhibited ICAM-1 and VCAM-1 expression in HUVECs, an effect associated with the high extract content of chlorogenic acid and anthocyanins, an aspect that was found to depend on the ripening state, harvest time, and geographical location, thus concluding that unripe berries may also have therapeutic potential [76].

Another study was performed on bovine coronary artery endothelial cells (BCAECs), where BCK juice induced a potent stimulatory effect on NO synthesis in coronary vessels. This aspect is related to endothelial nitric oxide synthase (eNOS) phosphorylation via redox-sensitive activation of the Src/PI3-kinase/Akt pathway (one of the primary mechanisms involved in NO synthesis), with conjugated cyanidins and chlorogenic acid being the main compounds responsible for this [77].

Further studies are required to fully understand BCK’s therapeutic potential, its mechanism of action, as well as its safety in cardiovascular pathogenicity.

## 4. Materials and Methods

### 4.1. Extracts’ Preparation, Reagents Used, and Phytochemical Characterization

The black chokeberry fruits and juice were sourced from the Aronia Charlottenburg Company in Timiș County, Romania, as noted in our previously published study [34]. The dried black chokeberries (kept at room temperature, approximately 23 °C, for one week) and the frozen black chokeberries (fresh fruits stored at −20 °C) were prepared at the Faculty of Pharmacy of “Victor Babeș” University of Medicine and Pharmacy of Timișoara (Discipline of Pharmacognosy, voucher specimen No. AM2/2022). A sample of each plant material (5 g) was finely chopped and combined with 10 mL of methanol, acidified with hydrochloric acid (0.3% *v*/*v*). The resulting mixture was subjected to ultrasonic treatment for 20 min at 35 °C and 40 kHz, using an ultrasonic bath. After this, the mixture was centrifuged (using a 320 Hettich centrifuge from Tuttlingen, Germany) at 5000 rpm (with a rotational radius of 10.5 cm and a relative centrifugal force of 2935) for 5 min, and the supernatant was collected. An additional 10 mL of solvent was added, and the centrifugation process was repeated. This procedure continued until the samples were colorless. The collected supernatant was then filtered under vacuum and concentrated to dryness using rotary evaporation at 35 °C. The resulting concentrated extract was placed in an oven at 35 °C for 4 days and subsequently stored at −20 °C until required [34].

The evaporated juice extract (EvArJ) was produced from 100% pure organic cold-pressed black chokeberry fruit juice purchased from the same company, as previously stated. Briefly, the BCK juice was derived from berries sourced from a certified organic plantation (BIO certification). The berries underwent automated harvesting, followed by a comprehensive washing procedure to eliminate surface contaminants. Subsequently, the berries were subjected to a pressing process to facilitate liquid extraction. The extracted juice was then filtered to remove solid particulates, ensuring clarity. To enhance microbial safety and extend shelf life, the juice underwent pasteurization. Following this treatment, the juice was bottled in sterilized, opaque containers to protect it from light exposure. The bottled juice was stored at room temperature, maintained below 23 °C, until the point of opening.

The EvArJ technique involved rotary evaporation at 35 °C, followed by drying in an oven at the same temperature for four days.

Additionally, the BCK extracts were dissolved in dimethyl sulfoxide (DMSO) (Sigma Aldrich, Saint Louis, MO, USA) to obtain a stock solution with a concentration of 100 mg/mL. Final solutions with concentrations of 1 μg/mL, 2.5 μg/mL, 5 μg/mL, 7.5 μg/mL, and 10 μg/mL were prepared by performing successive dilutions from intermediate concentrations of 500 μg/mL and 100 μg/mL in the culture medium.

### 4.2. EvArJ Characterization

The EvArJ extract’s composition was characterized in Cluj Napoca at the Faculty of Food Science and Technology, part of the University of Agricultural Science and Veterinary Medicine. The Agilent 1200 HPLC (high-performance liquid chromatography) system was employed, which contains an autosampler, solvent degasser, quaternary pump, and a UV–Vis (ultraviolet–visible) detector with a photodiode (DAD) coupled with an Agilent 6110 mass spectrometry (MS) single quadrupole detector (Agilent Technologies, Santa Clara, CA, USA). The components were separated using an Eclipse XDB C18 column with the dimensions of 4.6 × 150 mm, with 5 μm particles (Agilent Technologies, CA, USA). The two mobile phases, i.e., solvents A (0.1% aqueous acetic acid solution) and B (0.1% solution of acetic acid in acetonitrile), were utilized for a period of 30 min in the following gradient (expressed as % B), at 250 °C and with a flow rate of 0.5 mL/min: 0 min, 5% B; 0–2 min, 5% B; 2–18 min, 5–40% B; 18–20 min, 40–90% B; 20–24 min, 90% B; 24–25 min, 90%–5% B; and 25–30 min, 5% B. The chosen spectral range was 200–600 nm, so all chromatograms were recorded at the following wavelengths (λ): (a) 280 nm, (b) 340 nm, and (c) 520 nm. Regarding MS, the working mode involved the utilization of the ESI (electrospray ionization) positive ionization mode with the following settings: (1) temperature: 3000 °C; (2) capillary voltage: 3000 V; (3) m/z: 100–1200, full-scan; and (4) nitrogen flow: 8 l/min. The processing of the results was performed by using Agilent ChemStation software (Agilent Technologies, CA, USA). Firstly, qualitative analysis, consisting of identifying and assigning characteristic anthocyanin peaks, was based on comparing retention times and the ultraviolet–visible spectra with both standards and previously published data. Secondly, quantitative analysis was accomplished by using three different calibration curves. The phenolic acid content was quantified as chlorogenic acid equivalents, the flavonol content was quantified as rutin equivalents, and the anthocyanin content was quantified as cyanidin equivalents [34].

The phytochemical composition of the DryArs and the FrozArs is detailed in our already-published article [34].

### 4.3. Extracts’ Inorganic Element Detection (GF-AAS Method)

About 0.1 g of the dry extract was weighed out into glass capsules and was digested with 6 mL of 67% nitric acid (HNO_3_) (free of heavy metals), using a prescript program for food (P4) (MWS-2, Berghof system, Analytik Jena, Jena, Germany) and Teflon vessels (a DAP 60K pressure vessels, with an aluminum rupture disk, a TFM lid, and a coupling cap) [48].

After digestion, all samples were diluted to exactly 20 mL with ultrapure water. For each element, a calibration curve was achieved before measurement, using a special standard solution CertiPUR*, MERCK, 1000 mg/L, in HNO_3_ (e.g., Mangan–Standardlösung 1.19789.0100) (Table 6). The ultrapure water was obtained in the lab using the EASYpureRoDi–Barnstead apparatus (ThermoFisher Scientific, Waltham, MA, USA).

The technique used for determining heavy metals was atomic absorption spectrometry (AAS). A NovAA 400 apparatus (Analytik Jena, Jena, Germany) was used, equipped with a graphite furnace GF, a Cookbook for processing elements and samples, and a soft WinAAS 3.17.0 [48].

### 4.4. Cell Culture

The HPAECs were purchased from the American Type Culture Collection, ATCC (ATCC^®^ PCS-100-022) at passage 2 and grown according to the manufacturer’s indications, as follows: the cells (5000 cells/cm^2^) were grown in the basal medium vascular cell environment (ATCC, PCS-100-030), supplemented with the endothelial Cell Growth Kit-VEGF (ATCC, PCS-100-041) and a 1% antibiotic penicillin/streptomycin mixture (Sigma Aldrich, Saint Louis, MO, USA), and were maintained in incubators in an atmosphere of 37 °C, at 5% CO_2_. When the cells reached a confluence of about 80%, they were subcultivated for use for analysis: 5 mL of D-PBS (ATCC 30-2200) was added to remove serum residues, and the detachment of cells from the culture plate was achieved by adding 1 mL/25 cm^3^ of a preheated trypsin–EDTA (ethylenediaminetetraacetic acid) solution for primary cells (ATCC PCS-999-003). After detachment, an equal volume of trypsin neutralization solution from D-PBS (Dulbecco’s phosphate-buffered saline) (ATCC 30-2200) was added, supplemented with 5% fetal bovine serum (FBS, ATCC), was centrifuged at 1500 RPM (rotations per minute) for 5 min, and then was grown on the plates necessary for analysis.

### 4.5. In Vitro MTT Assay

Ten thousand cells/wells were sown on a culture plate with 96 wells and left for 24 h to attach the cells to the plate. The next day, the culture medium was removed, the final concentration test solutions were added (1 μg/mL, 2.5 μg/mL, 5 μg/mL, 7.5 μg/mL, and 10 μg/mL, obtained by successive dilutions of the stock solution in the culture medium), and then the cells were incubated for 72 h. After the incubation time had passed, the Cell Proliferation Kit I (MTT) (11465007001 Roche, Sigma Aldrich, Saint Louis, MO, USA) was used for cytotoxicity analysis, according to the manufacturer’s indications: a 10 μL/well MTT solution was added and cells were incubated for 3–4 h, after which a 100 μL/well solubilization solution was added, and the cells were reincubated for 30 min to 2 h for solubilization of the formed formazan salt. After solubilization, the samples were analyzed spectrophotometrically using a Tecan Infinite 200 Pro reader plate reader at a wavelength of 570 nm, with reference to 655 nm. Untreated cells and cells treated with pure solvent DMSO (0.01% equivalent to the highest contraction of DMSO present in the samples) were used as control samples. At this concentration, DMSO showed no significant action on the HPAECs. All experiments were conducted on microplates with 3 identical parallel wells. The results are presented as an average of 3 independent experiments ± the standard deviation. The statistical analysis was performed using a one-way ANOVA test in Microsoft Excel [77].

### 4.6. Cell Cycle Analysis

The cell cycle analysis was performed by means of flow cytometry. The cells, at a density of 2 × 10^5^ cells/well, were grown on 6-well plates for adherent cells (Greiner bio-one, Gmbh, Germany) and left for 24–28 h for attachment to the plate. Subsequently, the culture medium was removed, and a fresh medium containing the test extracts at final concentrations of 1 μg/mL and 10 μg/mL was added. The untreated cells were used as the control, and the cells treated with the solvent DMSO (0.01%) were used as the solvent control. After an incubation time of 72 h, the cells were trypsinized, collected, and fixed with 50% cold ethanol for a minimum of 30 min at 4 °C. After centrifugation (1500 rpm, 5 min, 22 °C), the cells were washed twice with D-PBS (ATCC 30-2200). Subsequently, 100 μL of propidium iodide (concentration, 100 μM) (Invitrogen, Carlsbad, CA, USA) was added for the marking of DNA (deoxyribonucleic acid) and the cells were incubated for 10 min in the dark. A fluorescence-activated cell sorter, the FACSCalibur flow meter (Becton-Dickinson, Franklin Lakes, NJ, USA), was used for data acquisition, and Flowing software 2.5.1 was used for the analysis of the cell cycle results [78].

### 4.7. Cell Death Analysis by Annexin V/PI Test

The evaluation of cell death induced by Aronia extracts on the HPAECs was analyzed by means of the Annexin V/PI method. Cells at a density of 2 × 105 cells/well were grown in 6-well plates for adherent cells (Greiner bio-one, Gmbh, Germany) and left for 24–28 h for attachment to the plate, and subsequently, the culture medium was removed, and a fresh medium containing the test extracts at final concentrations of 1 μg/mL and 10 μg/mL was added. The untreated cells were used as the control, and the DMSO solvent-treated cells (0.01%) were used as the solvent control. After an incubation time of 72 h, the cells were trypsinized, and the Annexin V-FITC kit combined with propidium iodide (PI) (Invitrogen, ThermoFisher, Vienna, Austria) was used in the cell death flow cytometric studies (apoptosis). After detachment from the culture plate, the cells were washed twice with the buffer solution 1 × Annexin V Binding Buffer, centrifuged at 1500 RPM for 5 min, resuspended in 195 μL of the buffer, and incubated with 5 μL of Annexin V-FITC for 15 min in the dark. After 15 min of incubation, the cells were washed with 200 μL of Annexin V Binding Buffer, resuspended in 190 μL of Annexin V Binding Buffer, and the propidium iodide solution was added at a volume of 10 μL 10 min before the flow cytometric analysis. The data were acquired with a FACSCalibur flow cytometer (Becton Dickinson, Franklin Lakes, NJ, United States), and the results were analyzed using the Flowing Software 2.5.1 program [79].

### 4.8. Statistical Analysis

The results are presented as an average of 3 independent experiments ± the standard deviation. A one-way ANOVA test, followed by Tukey’s test or Kruskal–Wallis with Dunn’s correction, was used to statistically determine the differences between experimental groups.

## 5. Conclusions

It is important to note that the reported results regarding the antiproliferative effects of BCKs, although modest, are preliminary. Further research is necessary to better understand the antiproliferative and/or cytotoxic effects of BCKs on non-pathological cell lines. Consequently, the data presented here will serve as a foundation for future studies, as there is limited information available from experiments involving cell lines, particularly in a cardiovascular context and with non-cancerous cell lines. The novelty of this study lies in the evaluation of BCK’s effects on the HPAEC line and the analysis of three different types of BCK extracts. Additionally, the identification of inorganic elements was conducted to enhance our understanding of the safety of these extracts. In conclusion, the available data support the potential of BCK as a nutrient-rich, health-promoting food with various benefits and functions.

## Figures and Tables

**Table 1:** The amounts of anthocyanins, hydroxycinnamic acids, and flavonols in the BCK samples, expressed as μg/g sample (LC-DAD-ESI-MS method).

	EvArJ (µg/g)	R_t_ (min)
Cy-3-*O*-diglucoside	0.03 C3GE	3.19
Neochlorogenic acid	**433.46** CCE	9.88
Cy-3-*O*-glucoside	**67.92** C3GE	10.98
Chlorogenic acid	**384.31** CCE	11.78
Cy-3-*O*-arabinoside	23.15 C3GE	11.96
Cy-3-*O*-xyloside	7.12 C3GE	12.13
Caffeic acid	30.58 CCE	13.27
Q-3-*O*-rutinoside (rutin)	41.24 RE	15.35
Q-3-*O*-glucoside	53.34 RE	16.16
Q	1.77 RE	21.71
**Total phenolic load**	**1042.93**	----

Legend: EvArJ—extract obtained from evaporated black chokeberry organic juice; CCE—chlorogenic acid equivalents; Cy—cyanidin; Q—quercetin; phenolic acids are expressed as chlorogenic acid equivalents (CCEs), flavonols are expressed as rutin equivalents (REs), and anthocyanins are expressed as cyanidin-3-*O*-glucoside equivalents (C3GEs). Bold formatting is used to draw attention to the most significant results.

**Table 2:** Metal concentrations (µg/g), as the mean of 3 determinations, in the DryArs, FrozArs, and EvArJ extracts.

	DryArs		FrozArs		EvArJ	
	Average	SV	Average	SV	Average	SV
As	*udl		*udl		*udl	
Al	277.355	9.05	329.037	22.52	380.039	15.99
Cd	0.049	0.00	0.026	0.01	0.043	0.01
Co	*udl		*udl		*udl	
Cr	1.135	0.01	0.275	0.08	1.375	0.02
Cu	0.343	0.06	0.282	0.05	0.243	0.07
Fe	396.728	15.75	302.667	20.56	597.665	30.21
Mn	1.418	0.12	1.177	0.23	1.550	0.13
Ni	*udl		*udl		*udl	
Pb	*udl		*udl		*udl	
Zn	44.344	5.53	58.909	9.47	44.708	4.36

Legend: *udl—under detection limit; SV—schedule variance; As—arsenic; Al—aluminum; Cd—cadmium; Co—cobalt; Cr—chromium; Cu—copper; Fe—iron; Mn—manganese; Ni—nickel; Pb—lead; Zn—zinc.

**Table 3:** Numerical data for the in vitro antiproliferative analysis of the BCK extracts on the HPAECs after 72 h of exposure to the selected samples using the MTT method, expressed as the % of cell viability.

	1 μg/mL	2.5 μg/mL	5 μg/mL	7.5 μg/mL	10 μg/mL
*DryArs*	63.55 ± 7.58	66.67 ± 2.45	62.58 ± 4.28	67.85 ± 4.57	65.91 ± 4.96
*FrozArs*	66.89 ± 0.63	65.57 ± 4.13	66.14 ± 5.64	66.38 ± 3.06	64.17 ± 1.46
*EvArJ*	67.60 ± 3.80	67.96 ± 5.85	67.63 ± 11.06	66.62 ± 7.54	72.57 ± 0.76

Legend: the results are presented as the mean ± SD of three different experiments.

**Table 4:** Results of cell cycle analysis on HPAECs after exposure for 72 h to BCK extracts.

	G0-G1%	S%	G2-M%	Sub-G0%
Control	62.66 ± 4.22	6.28 ± 1.50	21.56 ± 2.23	2.24 ± 1.38
DMSO 0.01%	61.08 ± 4.26	8.11 ± 2.72	19.41 ± 5.76	5.71 ± 4.13 *
*DryArs*—1 μg/mL	63.00 ± 4.69	7.80 ± 1.71	17.89 ± 2.61	6.56 ± 6.55
*DryArs*—10 μg/mL	57.14 ± 4.30 *	7.86 ± 1.06	16.85 ± 5.42 *	13.90 ± 8.67 **
*FrozArs*—1 μg/mL	62.12 ± 4.68	7.71 ±1.97	19.08 ± 1.00	5.46 ± 4.58
*FrozArs*—10 μg/mL	62.54 ± 2.66	6.79 ± 1.61	17.60 ± 4.31	8.18 ± 3.86
*EvArJ*—1 μg/mL	64.09 ± 3.52	8.05 ± 1.39	18.16 ± 2.06	2.56 ± 0.92
*EvArJ*—10 μg/mL	60.88 ± 3.96	7.54 ± 1.68	16.83 ± 3.35	9.76 ± 3.99

Observation: data = mean ± SEM, * *p* < 0.05, ** *p* < 0.01 vs. control (one-way ANOVA, Tukey’s test).

**Table 5:** Results of the Annexin V/PI analysis of the HPAECs after exposure for 72 h to the tested BCK extracts.

	Living Cells %	Cells in Early Apoptosis %	Apoptotic Cells %	Necrotic Cells %	Dying Cells %
Control	93.84 ± 0.77	1.86 ± 0.39	2.06 ± 0.52	2.23 ± 0.14	
DMSO 0.01%	93.39 ± 0.83	2.44 ± 0.14	2.16 ± 0.34	2.01 ± 0.63	
*DryArs*, 1 μg/mL	89.54 ± 2.27 *	1.42 ± 0.36	1.98 ± 0.36	7.06 ± 2.99 *	
*DryArs*, 10 μg/mL	80.82 ± 4.86 *	2.81 ± 0.33	3.27 ± 0.31 *	7.78 ± 4.20 **	5.30 ± 1.21%
*FrozArs*, 1 μg/mL	89.25 ± 2.05	3.17 ± 1.17	3.89 ± 1.48	3.69 ± 0.60	
*FrozArs*, 10 μg/mL	90.93 ± 1.39	2.40 ± 1.08	3.57 ± 1.59	3.10 ± 1.27	
*EvArJ,* 1 μg/mL	93.65 ± 1.52	2.28 ± 0.70	1.97 ± 0.65	2.11 ± 1.56	
*EvArJ*, 10 μg/mL	93.54 ± 0.98	1.39 ± 0.49	2.08 ± 0.24	2.98 ± 1.24	

Observation: data = mean ± SEM, * *p* < 0.05, ** *p* < 0.01 vs. control (one-way ANOVA, followed by Tukey’s test or Kruskal–Wallis with Dunn’s correction).

**Table 6:** Standard calibration curves for studied metals.

No	Metal	Wave, λ [nm]	Lower	Upper	Calibration Curve	R^2^
			Limit, µg/L	Limit, µg/L		
**1**	**As**	**193.7**	**13.2**	58.1	y = 0.00185 + 0.001544x	0.9927
2	Al	309.3	13.2	58.2	y = 0.006978 + 0.00175x	0.9971
3	Pb	283.3	7.4	37	y = 0.001778 + 0.003524x	0.9994
4	Cd	228.8	0.1	2.2	y = 0.004734 + 0.071971x	0.9923
5	Co	240.7	5.4	29.4	y = 0.008353 + 0.010864 x	0.9929
6	Cr	357.9	5	22	y = 0.018371 + 0.018435x	0.9961
7	Cu	324.8	3.6	18	y = 0.020731 + 0.016628 x	0.9961
8	Fe	248.3	3.6	14.4	y = 0.02274 + 0.013974	0.9939
9	Mn	297.5	0.84	4.2	y = 0.007792 + 0.112496x	0.9925
10	Ni	232	4.2	34.6	y = 0.033774 + 0.011603x	0.9967
11	Zn	213.9	1	8	y = 0.071658 + 0.092202x	0.9827

## Data Availability

The data are contained within this article.

## Abbreviations

The following abbreviations are used in this manuscript:

A-549	human lung adenocarcinoma
AAS	atomic absorption spectrometry
Al	aluminum
As	arsenic
ATCC	American Type Culture Collection
BCAECs	bovine coronary artery endothelial cells
BCK	black chokeberry
BJ-5ta	normal cell line
Caco-2	cell line of human colon cancer
CCE	chlorogenic acid equivalents
Cd	cadmium
Co	cobalt
Cr	chromium
Cu	cooper
Cy	cyanidin
C3GE	cyanidin-3-*O*-glucoside equivalent
CVD	cardiovascular disease
DAD	detector with a photodiode
DMSO	dimethyl sulfoxide
DNA	deoxyribonucleic acid
D-PBS	Dulbecco’s phosphate-buffered saline
DryArs	dry berries
ED	endothelial dysfunction
EDTA	ethylenediaminetetraacetic acid
EMA	European Medicines Agency
eNOS	endothelial nitric oxide synthase
ESI	electrospray ionization
EU	European Union
EvArJ	evaporated juice
FACS	fluorescence-activated cell sorter
FBS	fetal bovine serum
Fe	iron
FrozArs	frozen berries
GAE	gallic acid equivalent
GF	graphite furnace
H9c2	cell line of cardiomyoblasts
HDL	high-density lipoprotein
HeLa	human cervical adenocarcinoma
HNO_3_	nitric acid
HPAECs	human pulmonary artery endothelial cells
HPLC	high-performance liquid chromatography
HT-29	human colon cancer cells
HUVECs	human umbilical vein endothelial cells
IC50	half maximal inhibitory concentration
ICAM-1	intercellular adhesion molecule
ICP-OES	inductively coupled plasma optical emission spectroscopy technique
IL	interleukin
LC	lethal concentration
LC-DAD-ESI-MS	liquid chromatography coupled with diode array detection and electrospray ionization tandem mass spectrometry
LS-174T	human colorectal adenocarcinoma
mRNA	messenger ribonucleic acid
MCP-1	monocyte chemoattractant protein-1
MCF-7	breast cancer cell line
MCF-10A	normal cell line
MDA-MB-231	breast cancer cell line
Mn	manganese
MRC-5	normal lung fibroblasts
MS	mass spectrometry
NCM460	normal colon cells
Ni	nickel
NO	nitric oxide
OS	oxidative stress
p21WAF1	cyclin-dependent kinase inhibitor 1
p27KIP1	multifunctional cyclin-dependent kinase inhibitor with prognostic significance in human cancers
Pb	lead
PI	propidium iodide
PSA	potentiometric stripping analysis
Q	quercetin
RE	rutin equivalent
RMPI-1788	lymphoblast cell line
RPM	revolutions per minute
ROS	reactive oxygen species
SV	schedule variance
SV-HUC1	normal uroepithelial cell line
T24	urinary bladder cancer cell line
TIG-1	human lung embryonic fibroblasts
TNF-α	tumor necrosis factor-alpha
udl	under detection limit
UV-Vis	ultraviolet-visible
VCAM-1	vascular cell adhesion molecule
WHO	World Health Organization
WM793	melanoma cell line
Zn	zinc
786-O cancer cells	human renal cancer cell line

## References

[1] The Top 10 Causes of Death—WHO. https://www.who.int/news-room/fact-sheets/detail/the-top-10-causes-of-death.

[2] Di Cesare M., McGhie D.V., Perel P., Mwangi J., Taylor S., Pervan B., Narula J., Pineiro D., Pinto F.J. (2024). The Heart of the World. Glob. Heart.

[3] State of Health in the EU. Romania Country Health Profile 2023. https://www.oecd.org/en/publications/romania-country-health-profile-2023_f478769b-en.html.

[4] Man A.W.C., Li H., Xia N. (2020). Impact of Lifestyles (Diet and Exercise) on Vascular Health: Oxidative Stress and Endothelial Function. Oxidative Med. Cell. Longev..

[5] Theodoridis X., Chourdakis M., Papaemmanouil A., Chaloulakou S., Georgakou A.V., Chatzis G., Triantafyllou A. (2024). The Effect of Diet on Vascular Aging: A Narrative Review of the Available Literature. Life.

[6] Li A., Yan J., Zhao Y., Yu Z., Tian S., Khan A.H., Zhu Y., Wu A., Zhang C., Tian X.L. (2023). Vascular Aging: Assessment and Intervention. Clin. Interv. Aging.

[7] Biswas I., Khan G.A., Shad K.F., Saravi S.S.S., Bilgrami N.L. (2020). Endothelial Dysfunction in Cardiovascular Diseases. Basic and Clinical Understanding of Microcirculation.

[8] Buda V., Andor M., Diana A., Ardelean F., Zinuca Pavel I., Dehelean C., Soica C., Folescu R., Andrei F., Danciu C., Sharma K., Mishra K., Senarpati K.K., Danciu C. (2021). Cardioprotective Effects of Cultivated Black Chokeberries (*Aronia* spp.): Traditional Uses, Phytochemistry and Therapeutic Effects. Bioactive Compounds in Nutraceutical and Functional Food for Good Human Health.

[9] Bays H.E., Taub P.R., Epstein E., Michos E.D., Ferraro R.A., Bailey A.L., Kelli H.M., Ferdinand K.C., Echols M.R., Weintraub H. (2021). Ten things to know about ten cardiovascular disease risk factors. Am. J. Prev. Cardiol..

[10] Christ A., Lauterbach M., Latz E. (2019). Western Diet and the Immune System: An Inflammatory Connection. Immunity.

[11] Kapoor G., Chauhan P., Singh G., Malhotra N., Chahal A. (2022). Physical Activity for Health and Fitness: Past, Present and Future. J. Lifestyle Med..

[12] Almarshad M.I., Algonaiman R., Alharbi H.F., Almujaydil M.S., Barakat H. (2022). Relationship between Ultra-Processed Food Consumption and Risk of Diabetes Mellitus: A Mini-Review. Nutrients.

[13] Zhang Y.B., Pan X.F., Chen J., Cao A., Xia L., Zhang Y., Wang J., Li H., Liu G., Pan A. (2021). Combined lifestyle factors, all-cause mortality and cardiovascular disease: A systematic review and meta-analysis of prospective cohort studies. J. Epidemiol. Community Health.

[14] Nyulas K.I., Simon-Szabó Z., Pál S., Fodor M.A., Dénes L., Cseh M.J., Barabás-Hajdu E., Csipor B., Szakács J., Preg Z. (2024). Cardiovascular Effects of Herbal Products and Their Interaction with Antihypertensive Drugs—Comprehensive Review. Int. J. Mol. Sci..

[15] Cena H., Calder P.C. (2020). Defining a healthy diet: Evidence for the role of contemporary dietary patterns in health and disease. Nutrients.

[16] Mishra T., Kondepati A.K., Pasumarthi S.D., Chilana G.S., Devabhaktuni S., Singh P.K. (2020). Phytotherapeutic antioxidants. Asian J. Med. Sci..

[17] Mahomoodally M.F., Mooroteea K. (2021). A comparative ethno-religious study of traditionally used medicinal plants employed in the management of cardiovascular diseases. J. Herb. Med..

[18] Saracila M., Untea A.E., Oancea A.G., Varzaru I., Vlaicu P.A. (2024). Comparative Analysis of Black Chokeberry (*Aronia melanocarpa* L.) Fruit, Leaves, and Pomace for Their Phytochemical Composition, Antioxidant Potential, and Polyphenol Bioaccessibility. Foods.

[19] Sapian S., Taib I.S., Katas H., Latip J., Zainalabidin S., Hamid Z.A., Anuar N.N.M., Budin S.B. (2022). The Role of Anthocyanin in Modulating Diabetic Cardiovascular Disease and Its Potential to Be Developed as a Nutraceutical. Pharmaceuticals.

[20] Panchal S.K., John O.D., Mathai M.L., Brown L. (2022). Anthocyanins in Chronic Diseases: The Power of Purple. Nutrients.

[21] Mattioli R., Francioso A., Mosca L., Silva P. (2020). Anthocyanins: A Comprehensive Review of Their Chemical Properties and Health Effects on Cardiovascular and Neurodegenerative Diseases. Molecules.

[22] Nistor M., Pop R., Daescu A., Pintea A., Socaciu C., Rugina D. (2022). Anthocyanins as Key Phytochemicals Acting for the Prevention of Metabolic Diseases: An Overview. Molecules.

[23] Carvalho F., Lahlou R.A., Silva L.R. (2024). Phenolic Compounds from Cherries and Berries for Chronic Disease Management and Cardiovascular Risk Reduction. Nutrients.

[24] Ngamsamer C., Sirivarasai J., Sutjarit N. (2022). The Benefits of Anthocyanins against Obesity-Induced Inflammation. Biomolecules.

[25] Ren Y., Frank T., Meyer G., Lei J., Grebenc J.R., Slaughter R., Gao Y.G., Kinghorn A.D. (2022). Potential Benefits of Black Chokeberry (*Aronia melanocarpa*) Fruits and Their Constituents in Improving Human Health. Molecules.

[26] Jurendi’c T.J., Ščetar M., Voilley A., Kurek M. (2021). *Aronia melanocarpa* Products and By-Products for Health and Nutrition: A Review. Antioxidants.

[27] Shi D., Xu J., Sheng L., Song K. (2024). Comprehensive Utilization Technology of *Aronia melanocarpa*. Molecules.

[28] Staszowska-Karkut M., Materska M. (2020). Phenolic composition, mineral content, and beneficial bioactivities of leaf extracts from black currant (*Ribes nigrum* L.), raspberry (*Rubus idaeus*), and aronia (*Aronia melanocarpa*). Nutrients.

[29] Sidor A., Gramza-Michałowska A. (2019). Black Chokeberry *Aronia melanocarpa* L.—A Qualitative Composition, Phenolic Profile and Antioxidant Potential. Molecules.

[30] Go M.Y., Kim J., Jeon C.Y., Shin D.W. (2024). Functional Activities and Mechanisms of *Aronia melanocarpa* in Our Health. Curr. Issues Mol. Biol..

[31] Avula B., Katragunta K., Osman A.G., Ali Z., John Adams S., Chittiboyina A.G., Khan I.A. (2023). Advances in the Chemistry, Analysis and Adulteration of Anthocyanin Rich-Berries and Fruits: 2000–2022. Molecules.

[32] Gerasimov M.A., Perova I.B., Eller K.I., Akimov M.Y., Sukhanova A.M., Rodionova G.M., Ramenskaya G.V. (2023). Investigation of Polyphenolic Compounds in Different Varieties of Black Chokeberry *Aronia melanocarpa*. Molecules.

[33] Olechno E., Puścion-Jakubik A., Zujko M.E. (2022). Chokeberry (*A. melanocarpa* (Michx.) Elliott)—A Natural Product for Metabolic Disorders?. Nutrients.

[34] Buda V., Sturza A., Minda D., Diaconeasa Z., Iuhas C., Bădescu B., Dehelean C.A., Danciu C., Muntean M.D., Lighezan R. (2024). Vasculo-Protective Effects of Standardized Black Chokeberry Extracts in Mice Aorta. Int. J. Mol. Sci..

[35] Romania: Country Health Profile 2021. https://www.oecd.org/en/publications/romania-country-health-profile-2021_74ad9999-en.html.

[36] Causes of Death Statistics. https://ec.europa.eu/eurostat/statistics-explained/index.php?title=Causes_of_death_statistics.

[37] Kaloudi T., Tsimogiannis D., Oreopoulou V. (2022). Aronia Melanocarpa: Identification and Exploitation of Its Phenolic Components. Molecules.

[38] Niesen S., Göttel C., Becker H., Bakuradze T., Winterhalter P., Richling E. (2022). Fractionation of Extracts from Black Chokeberry, Cranberry, and Pomegranate to Identify Compounds That Influence Lipid Metabolism. Foods.

[39] Gao N., Shu C., Wang Y., Tian J., Lang Y., Jin C., Cui X., Jiang H., Liu S., Li Z. (2024). Polyphenol components in black chokeberry (*Aronia melanocarpa*) as clinically proven diseases control factors—An overview. Food Sci. Hum. Wellness.

[40] Vagiri M., Jensen M. (2017). Influence of juice processing factors on quality of black chokeberry pomace as a future resource for colour extraction. Food Chem..

[41] Salazar-Orbea G.L., García-Villalba R., Bernal M.J., Hernández-Jiménez A., Egea J.A., Tomás-Barberán F.A., Sánchez-Siles L.M. (2023). Effect of Storage Conditions on the Stability of Polyphenols of Apple and Strawberry Purees Produced at Industrial Scale by Different Processing Techniques. J. Agric. Food Chem..

[42] Torović L., Sazdanić D., Krstonošić M.A., Mikulić M., Beara I., Cvejić J. (2023). Compositional characteristics, health benefit and risk of commercial bilberry and black chokeberry juices. Food Biosci..

[43] Pop L., Costa R., Asănică A., Tudoreanu L. (2022). Mineral nutritional value of products containing aronia fruits and juices: A review. Horticulture.

[44] Pavlovic A.N., Brcanovic J.M., Veljkovic J.N., Mitic S.S., Tošic S.B., Kaličanin B.M., Kostić D.A., Dordević M.S., Velimirović D.S. (2015). Characterization of commercially available products of aronia according to their metal content. Fruits.

[45] Kaličanin B., Pavlović A., Velimirović D., Arsić I., Dordević S., Tadić V. (2020). Optimization and Application of Potentiometric Stripping Analysis for Determination of Heavy Metals in the samples of *Aronia melanocarpa* (Michx.) Elliot. Int. J. Electrochem. Sci..

[46] Mężyńska M., Brzóska M.M., Rogalska J., Piłat-Marcinkiewicz B. (2019). Extract from aronia melanocarpa l. Berries prevents cadmium-induced oxidative stress in the liver: A study in a rat model of low-level and moderate lifetime human exposure to this toxic metal. Nutrients.

[47] de Abreu C.B., de ORibeiro M., Pinho C.S., Carneiro C.N., de Azevedo Neto A.D., de Souza M.O., de S Dias F. (2021). Exploratory analysis in the evaluation of stress due to aluminum presence in *Physalis angulata* L. and multielement determination by microwave-induced plasma optical emission spectrometry (MIP OES). Environ. Sci. Pollut..

[48] Kis B., Pavel I.Z., Haidu D., Ștefănuț M.N., Diaconeasa Z., Moacă E.A., Dehelean C.A., Șipos S., Ivan A., Danciu C. (2021). Inorganic Element Determination of Romanian *Populus nigra* L. Buds Extract and In Vitro Antiproliferative and Pro-Apoptotic Evaluation on A549 Human Lung Cancer Cell Line. Pharmaceutics.

[49] ICH Q3D Elemental Impurities—Scientific Guideline. https://www.ema.europa.eu/en/ich-q3d-elemental-impurities-scientific-guideline.

[50] WHO Guidelines for Assessing Quality of Herbal Medicines with Reference to Contaminants and Residues. https://iris.who.int/handle/10665/43510.

[51] European Food Safety Authority (EFSA) (2008). Safety of Aluminium from Dietary Intake—Scientific Opinion of the Panel on Food Additives, Flavourings, Processing Aids and Food Contact Materials (AFC). EFSA J..

[52] Haidu D., Párkányi D., Moldovan R.I., Savii C., Pinzaru I., Dehelean C., Kurunczi L. (2017). Elemental Characterization of Romanian Crop Medicinal Plants by Neutron Activation Analysis. J. Anal. Methods Chem..

[53] Tietz T., Lenzner A., Kolbaum A.E., Zellmer S., Riebeling C., Gürtler R., Jung C., Kappenstein O., Tentschert J., Giulbudagian M. (2019). Aggregated Aluminium Exposure: Risk Assessment for the General Population. Arch. Toxicol..

[54] Cindrić I.J., Zeiner M., Mihajlov-Konanov D., Stingeder G. (2017). Inorganicmacro- andmicronutrients in “superberries” black chokeberries (*Aronia melanocarpa*) and related teas. Int. J. Environ. Res. Public Health.

[55] Olechno E., Puścion-Jakubik A., Soroczyńska J., Socha K., Zujko M.E. (2023). Are Chokeberry Products Safe for Health? Evaluation of the Content of Contaminants and Health Risk. Foods.

[56] Aksoy A.S. (2023). A Review of the Nutritional Profile, Chemical Composition and Potential Health Benefits of *Aronia melanocarpa* (Chokeberry) Berries and Products. Turk. J. Agric.—Food Sci. Technol..

[57] Malik M., Zhao C., Schoene N., Guisti M.M., Moyer M.P., Magnuson B.A. (2003). Anthocyanin-Rich Extract from *Aronia meloncarpa* E. Induces a Cell Cycle Block in Colon Cancer but Not Normal Colonic Cells. Nutr. Cancer.

[58] Gill N.K., Rios D., Osorio-Camacena E., Mojica B.E., Kaur B., Soderstrom M.A., Gonzalez M., Plaat B., Poblete C., Kaur N. (2021). Anticancer Effects of Extracts from Three Different Chokeberry Species. Nutr. Cancer.

[59] Dvorska D., Mazurakova A., Lackova L., Sebova D., Kajo K., Samec M., Brany D., Svajdlenka E., Treml J., Mersakova S. (2024). *Aronia melanocarpa* L. fruit peels show anti-cancer effects in preclinical models of breast carcinoma: The perspectives in the chemoprevention and therapy modulation. Front Oncol..

[60] Bermúdez-Soto M.J., Larrosa M., Garcia-Cantalejo J.M., Espín J.C., Tomás-Barberan F.A., García-Conesa M.T. (2007). Up-regulation of tumor suppressor carcinoembryonic antigen-related cell adhesion molecule 1 in human colon cancer Caco-2 cells following repetitive exposure to dietary levels of a polyphenol-rich chokeberry juice. J. Nutr. Biochem..

[61] Sharif T., Stambouli M., Burrus B., Emhemmed F., Dandache I., Auger C., Etienne-Selloum N., Schini-Kerth V.B., Furhrmann G. (2013). The polyphenolic-rich *Aronia melanocarpa* juice kills teratocarcinomal cancer stem-like cells, but not their differentiated counterparts. J. Funct. Foods.

[62] Nowak D., Kloskowski T., Gośliński M., Buhl M., Wojtowicz E., Popławski C., Drewa T., Pokrywczyńska M. (2025). Antioxidant Properties of *Aronia melanocarpa* and Morinda citrifolia Juices and their Impact on Bladder Cancer Cell Lines. Med. Sci. Monit..

[63] Borczak B., Kapusta-Duch J., Domagała D., Doskočil I. (2023). Study on the Potential Antitumor Activity of Cookies Enriched with *Sambucus nigra* L., *Aronia melanocarpa*, *Hippophae rhamnoides* L., and *Crataegus* L., on WM793 Melanoma and MCF-7 Breast Cell Lines. Appl. Sci..

[64] Bushmeleva K., Vyshtakalyuk A., Terenzhev D., Belov T., Nikitin E., Zobov V. (2023). *Aronia melanocarpa* Flavonol Extract—Antiradical and Immunomodulating Activities Analysis. Plants.

[65] Matsuo M., Sasaki N., Saga K., Kaneko T. (2005). Cytotoxicity of flavonoids toward cultured normal human cells. Biol. Pharm. Bull..

[66] Cvetanović A., Zengin G., Zeković Z., Švarc-Gajić J., Ražić S., Damjanović A., Mašković P., Mitić M. (2018). Comparative in vitro studies of the biological potential and chemical composition of stems, leaves and berries *Aronia melanocarpa*’s extracts obtained by subcritical water extraction. Food Chem. Toxicol..

[67] Caparica R., Júlio A., Araújo M.E.M., Baby A.R., Fonte P., Costa J.G., Santos de Almeida T. (2020). Anticancer activity of rutin and its combination with ionic liquids on renal cells. Biomolecules.

[68] Hidalgo M., Sánchez-Moreno C., de Pascual-Teresa S. (2010). Flavonoid-flavonoid interaction and its effect on their antioxidant activity. Food Chem..

[69] Joshi T., Deepa P.R., Sharma P.K. (2022). Effect of Different Proportions of Phenolics on Antioxidant Potential: Pointers for Bioactive Synergy/Antagonism in Foods and Nutraceuticals. Proc. Natl. Acad. Sci. India Sect. B—Biol. Sci..

[70] Mladěnka P., Zatloukalová L., Šimůnek T., Bobrovová Z., Semecký V., Nachtigal P., Hašková P., Macková E., Vávrová J., Holečková M. (2009). Direct administration of rutin does not protect against catecholamine cardiotoxicity. Toxicology.

[71] Filipský T., Říha M., Hašková P., Pilařová V., Nováková L., Semecký V., Vávrová J., Holečková M., Palicka V., Šimůnek T. (2017). Intravenous rutin in rat exacerbates isoprenaline-induced cardiotoxicity likely due to intracellular oxidative stress. Redox Rep..

[72] Borecki K., Żuchowski M., Siennicka A., Adler G., Jastrzębska M. (2016). Polyphenol rich extract of *Aronia melanocarpa* inhibits TNF α induced apoptosis in H9c2 cells. J. Med. Sci..

[73] Zapolska-Downar D., Bryk D., Małecki M., Hajdukiewicz K., Sitkiewicz D. (2012). *Aronia melanocarpa* fruit extract exhibits anti-inflammatory activity in human aortic endothelial cells. Eur. J. Nutr..

[74] Luzak B., Golanski J., Rozalski M., Krajewska U., Olas B., Watala C. (2010). Extract from *Aronia melanocarpa* fruits potentiates the inhibition of platelet aggregation in the presence of endothelial cells. Arch. Med. Sci..

[75] Iwashima T., Kudome Y., Kishimoto Y., Saita E., Tanaka M., Taguchi C., Hirakawa S., Mitani N., Kondo K., Iida K. (2019). Aronia berry extract inhibits TNF-α-induced vascular endothelial inflammation through the regulation of STAT3. Food Nutr. Res..

[76] Zielińska A., Bryk D., Paradowska K., Siudem P., Wawer I., Wrzosek M. (2024). Anti-Atherosclerotic Properties of *Aronia melanocarpa* Extracts Influenced by Their Chemical Composition Associated with the Ripening Stage of the Berries. Int J Mol Sci..

[77] Kim J.H., Choi M.S., Auger C., Lee K.W., Schini-Kerth V.B. (2024). Polyphenol-rich *Aronia melanocarpa* juice sustains eNOS activation through phosphorylation and expression via redox-sensitive pathways in endothelial cells. Food Sci. Biotechnol..

[78] Oprean C., Bojin F., Soica C., Drăghia L., Caunii A., Paunescu V., Tatu C. (2018). Selective in vitro anti-melanoma activity of ursolic and oleanolic acids. Toxicol. Mech. Methods.

[79] Ungureanu A.R., Popovici V., Oprean C., Danciu C., Schröder V., Olaru O.T., Mihai D.P., Popescu L., Luță E.A., Chițescu C.L. (2023). Cytotoxicity Analysis and In Silico Studies of Three Plant Extracts with Potential Application in Treatment of Endothelial Dysfunction. Pharmaceutics.

